# Guideline Concordance and Documentation of Allopurinol Initiation Across Clinical Specialties: A Three-Year Retrospective Evaluation

**DOI:** 10.3390/healthcare14203454

**Published:** 2026-10-15

**Authors:** Aos Aboabat, Mohamed Bedaiwi, Roqayah Abdullah Almaradheef, Abdullah Aldhuwaihy, Rakan Alarifi, Waleed F. Alanazi, Ziyad B. Alenazi, Mohanned Alessa, Hatem Alnasser, Sarah Sewaralthahab, Ibrahim Almaghlouth, Abdurhman S. Alarfaj, Haya M. Almalag

**Affiliations:** 1 Rheumatology Unit, Department of Medicine, King Saud University, Riyadh 11451, Saudi Arabia; mbedaiwi@ksu.edu.sa (M.B.);; 2 College of Medicine, King Saud University, Riyadh 11451, Saudi Arabia; 3 Urology Unit, Department of Surgery, King Saud University, Riyadh 11451, Saudi Arabia; 4 Nephrology Unit, Department of Medicine, King Saud University, Riyadh 11451, Saudi Arabia; 5 Hematology Unit, Department of Medicine, King Saud University, Riyadh 11451, Saudi Arabia; 6 Department of Clinical Pharmacy, College of Pharmacy, King Saud University, Riyadh 11451, Saudi Arabia

**Keywords:** allopurinol, guideline concordance, urate-lowering therapy, prescribing quality, hyperuricemia, medication safety

## Abstract

**Background/Objectives:** To assess guideline concordance and indication documentation of new allopurinol prescriptions and whether these differed across clinical specialties. **Methods:** A retrospective study at a tertiary academic center in Riyadh, Saudi Arabia, included adults with a first institutional allopurinol dispensation between January 2022 and November 2024. Prescriptions were guideline-concordant if issued for gout meeting 2020 American College of Rheumatology initiation criteria, tumor lysis syndrome management, or recurrent nephrolithiasis; documented non-guideline-concordant if another indication was recorded; and undocumented otherwise. Specialty associations were assessed using logistic regression, age- and sex-adjusted Firth models, and Fisher’s exact tests. **Results:** Among 707 patients, 105 (14.9%) prescriptions were guideline-concordant, 452 (63.9%) were documented non-guideline-concordant, and 150 (21.2%) were undocumented; asymptomatic hyperuricemia accounted for 47.0%. Relative to Rheumatology, Primary Care (OR 92.57, 95% CI 32.89–260.57) and Nephrology (OR 24.69, 95% CI 7.01–86.90) had higher odds of documented non-concordance, persisting after adjustment. Cardiology had the highest undocumented rate (35.4%). **Conclusions:** Non-guideline-concordant initiation was common, and the type of quality gap differed by specialty. These observational associations may partly reflect case mix and should not be attributed to specialty alone, but they support specialty-tailored stewardship.

## 1. Introduction

Hyperuricemia is common, affecting more than 20% of men and approximately 4% of women in the general population [1]. In Saudi Arabia, an early prospective survey of primary care attendees reported a hyperuricemia prevalence of 8.4% [2]. However, most individuals with hyperuricemia never develop gout: even among those with serum urate ≥10 mg/dL, fewer than half develop gout over 15 years [1], and among those with serum urate >9 mg/dL, approximately 20% develop gout within 5 years [3].

Urate-lowering therapy (ULT) is recommended for carefully selected patient populations. Current guidelines endorse ULT initiation primarily in patients with gout who meet specific clinical criteria, including recurrent flares, tophaceous disease, radiographic damage attributable to gout, or very high serum urate levels, as outlined in the 2020 American College of Rheumatology (ACR) guideline, which conditionally recommends against ULT for asymptomatic hyperuricemia [3]. Beyond gout, guideline-supported uses of allopurinol include selected patients with uric acid or calcium oxalate nephrolithiasis and hyperuricosuria, and tumor lysis syndrome (TLS) prophylaxis or management in appropriate oncologic settings [4,5,6,7]. In contrast, asymptomatic hyperuricemia, defined as elevated serum urate levels without prior gout flares or subcutaneous tophi, does not constitute an indication for ULT. The role of ULT in patients with chronic kidney disease (CKD) without gout has also been clarified. Although observational data and small trials had suggested that urate lowering might slow CKD progression [8], a randomized trial of febuxostat in stage 3 CKD with asymptomatic hyperuricemia showed no renal benefit [9], and two adequately powered randomized controlled trials (RCTs) published in 2020 found no renal benefit from allopurinol in patients with CKD or type 1 diabetic kidney disease [10,11]. The 2024 Kidney Disease: Improving Global Outcomes (KDIGO) guideline also suggests against urate-lowering therapy to delay CKD progression in people with asymptomatic hyperuricemia [12]. Together, these data support the guideline recommendations against routine ULT use in asymptomatic individuals, emphasizing clinical manifestations rather than serum urate concentrations alone.

Non-guideline-concordant or undocumented ULT prescribing represents an important quality-of-care gap, with implications for patient safety, healthcare utilization, and guideline adherence. This is particularly relevant given that allopurinol is associated with potentially serious adverse effects, including severe cutaneous adverse reactions. Allopurinol is the most common cause of Stevens–Johnson syndrome/toxic epidermal necrolysis in Europe [13]; mortality from allopurinol hypersensitivity syndrome ranges from 9% to 20% [14,15]; and in a nationwide cohort of new allopurinol users, 8.3% of patients who developed allopurinol hypersensitivity died, with higher risk when allopurinol was used for asymptomatic hyperuricemia with renal or cardiovascular disease [16].

Previous Saudi studies have already demonstrated substantial allopurinol overuse. An early tertiary-center audit found that only 29.5% of prescriptions had an appropriate indication [17], and subsequent studies reported that 77.8% of allopurinol-treated adults [18] and 76.4% of those with CKD [19] lacked a valid indication. Surveys of Saudi physicians describe uncertainty about urate thresholds and the distinction between asymptomatic hyperuricemia and gout [20,21,22], with the lowest knowledge scores among general practitioners [23], suggesting that prescribing practices may reflect differences in training, familiarity with current evidence, and the clinical problems each specialty manages. However, these studies were descriptive, included prevalent allopurinol use, applied pre-2020 criteria that considered marked asymptomatic hyperuricemia a potentially valid indication, and did not characterize how different prescribing-quality failures vary across clinical specialties.

Interventions to improve prescribing quality for other medicines provide a framework for addressing these gaps. Clinical decision support integrated into electronic medical records has produced modest reductions in potentially inappropriate prescribing, most consistently in hospital settings [24], and systematic capture of the treatment indication at the time of prescribing is feasible and accurate [25]. The appropriate intervention depends on the nature of the gap: a documented decision to treat an unsupported indication calls for evidence-focused education and decision support, whereas prescribing without a recorded rationale first requires reliable capture of the indication.

A prior analysis from our institution examined the subgroup of new allopurinol users with a physician-documented diagnosis of gout [26]. The present study addresses a separate question by examining the full spectrum of new allopurinol prescribing across the health system.

The persistence of allopurinol prescribing outside contemporary evidence-based indications raises an implementation question that has received less attention than the overall prevalence of overprescribing: whether the type of prescribing-quality gap differs across clinical specialties. We therefore evaluated new allopurinol prescriptions across an academic health system using a prespecified contemporary guideline framework, distinguishing documented non-guideline-concordant prescribing from absent indication documentation and examining how these patterns differed across prescribing specialties. The objective was to identify specialty-specific targets for prescribing improvement and medication safety.

## 2. Materials and Methods

### 2.1. Study Design and Setting

We conducted a retrospective, criteria-based evaluation of allopurinol prescribing at King Saud University Medical City (KSUMC), a tertiary academic hospital in Riyadh, Saudi Arabia, which serves as the principal teaching hospital of King Saud University and provides primary, specialty, and subspecialty ambulatory care across multiple clinical departments. KSUMC operates a unified electronic medical record (EMR) across ambulatory, emergency, and inpatient services, permitting capture of prescribing and clinical documentation across settings. The study period extended from 1 January 2022 to 30 November 2024. No changes to national regulatory requirements or to institutional prescribing policies for allopurinol occurred during the study period. Eligible patients were identified using institutional pharmacy dispensing records. The study was reported in accordance with the Strengthening the Reporting of Observational Studies in Epidemiology (STROBE) guidelines.

### 2.2. Study Population

**Figure 1:**
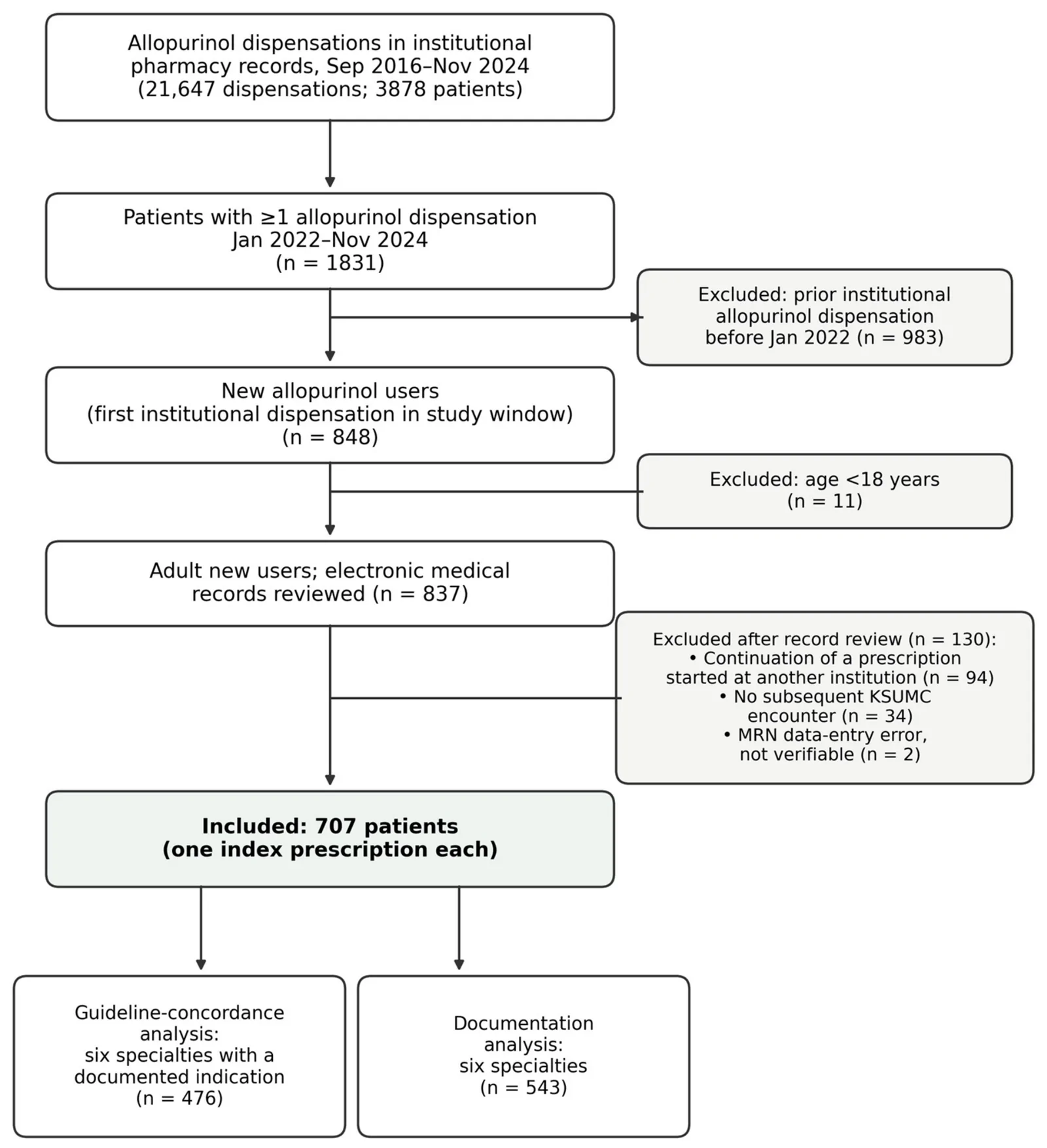
Flow diagram of patient selection. Descriptive analyses included all 707 patients. Specialty-level inferential analyses were restricted to the six specialties with ≥20 prescriptions (Primary Care, Hematology/Oncology, Cardiology, Nephrology, Rheumatology, and Urology). KSUMC = King Saud University Medical City; MRN = medical record number.

Eligible patients were identified from institutional pharmacy dispensing records. Inclusion criteria were: (1) age ≥18 years at the index dispensation; (2) a new allopurinol prescription during the study period; and (3) at least one subsequent KSUMC encounter after the index prescription, ensuring sufficient longitudinal record availability to review documentation relevant to the index allopurinol prescription. A “new prescription” was defined as an allopurinol dispensation with no prior allopurinol dispensation recorded in the institutional pharmacy system. Pharmacy records were available from September 2016, providing a look-back period of at least five years for every patient. Exclusion criteria were: (1) documentation in the EMR that the prescription was a continuation of allopurinol initiated at another institution; (2) no subsequent KSUMC encounter; and (3) records whose medical record number could not be verified. Each patient contributed one index prescription. Screening was performed in two stages: pharmacy dispensing records were first used to identify patients whose first institutional allopurinol dispensation occurred within the study period, after which the EMR of each remaining adult patient was reviewed manually by the study team to confirm eligibility. The selection process is shown in Figure 1.

### 2.3. Data Sources and Variables

Institutional pharmacy dispensing records were used to identify all eligible patients, and a structured review of the EMR was performed for each patient to extract the prescribing physician’s specialty, documented indication, and International Classification of Diseases (ICD) codes. Age and sex at the index dispensation were obtained from institutional pharmacy records. Patients with physician-documented gout in the present cohort were previously reported in an analysis focused on gout-specific quality indicators and subsequent gout management [26].

### 2.4. Indication Classification

The documented indications for each prescription were ascertained from the structured EMR fields, active problem lists, ICD codes, and physician notes. Where no indication was coded or documented in the clinical record, the prescription was classified as “indication not documented.” Patients with a diagnosis of gout were those with an explicit physician-recorded diagnosis in the EMR, irrespective of the specialty or diagnostic method (clinical, crystal-confirmed, or imaging-based) used. Each prescription was assigned to one of six indication categories: (1) physician-documented gout, (2) asymptomatic hyperuricemia, (3) CKD without gout, (4) TLS management, (5) recurrent uric acid or calcium oxalate nephrolithiasis, and (6) undocumented indications. No hierarchy was required to determine guideline concordance: a prescription was classified as guideline-concordant if at least one guideline-concordant indication was documented, irrespective of coexisting diagnoses, and no prescription in this cohort had more than one guideline-concordant indication. All patients in the CKD-without-gout category also had hyperuricemia; when CKD was documented as the reason for treatment, the prescription was assigned to the CKD category rather than to asymptomatic hyperuricemia. The asymptomatic hyperuricemia category was operationally defined as prescriptions in which the clinical records, including physician notes, problem lists, and ICD codes, reflected the isolated elevation of serum urate as the prescribing context in the absence of a physician-documented gout diagnosis, documented clinical features, or any other recognized indication, and required a serum urate >6.8 mg/dL, consistent with the ACR 2020 definition of asymptomatic hyperuricemia [3]. CKD without gout was defined as a prescription for which CKD was documented by the prescriber as the reason for allopurinol, in a patient without physician-documented gout or another guideline-concordant indication; this category was based on the documented indication rather than on an estimated glomerular filtration rate threshold. Prescribers did not uniformly use the term “asymptomatic hyperuricemia” in their documentation; the classification represented an investigator-assigned categorization based on the totality of available clinical record data.

### 2.5. Guideline-Concordance Framework

Prescriptions were classified using a prespecified three-category framework based on contemporary guideline recommendations and trial evidence. A prescription was classified as guideline-concordant if it met one of the following criteria: (1) physician-documented gout meeting at least one 2020 ACR criterion for ULT initiation, including subcutaneous tophi, radiographic gout damage, ≥2 documented flares, or a first flare accompanied by CKD stage ≥3, serum urate >9 mg/dL, or urolithiasis [3]; (2) tumor lysis syndrome management, defined as allopurinol initiated for TLS prophylaxis or treatment in patients receiving anticancer therapy according to the 2023 United States expert consensus guideline, which recommends allopurinol prophylaxis across low-, intermediate-, and high-risk categories and allopurinol for adults with established laboratory or clinical TLS [6]; or (3) prevention of recurrent nephrolithiasis was considered an appropriate indication for allopurinol in patients with recurrent stone disease who met either of the following criteria: (a) calcium oxalate nephrolithiasis with hyperuricosuria (>4.0 mmol/day), or uric acid or ammonium urate nephrolithiasis in hyperuricosuric stone formers (>4.0 mmol/day), in accordance with the European Association of Urology guideline [7]; or (b) active recurrent nephrolithiasis despite adequate fluid intake, for which pharmacologic preventive therapy with a thiazide diuretic, citrate, or allopurinol is recommended by the American College of Physicians guideline [5]. Flare history was abstracted as the number of gout flares documented in the EMR before allopurinol initiation. Because both the strong ACR recommendation (≥2 flares per year) and the conditional recommendation (more than one previous flare with infrequent flares) support ULT initiation, patients with two or more documented flares were classified as meeting the initiation criteria irrespective of annual flare frequency; patients with a single documented flare were assessed against the first-flare criteria. When flare history was not documented, patients were conservatively classified as not meeting flare-based criteria [26]. Prescriptions with a documented indication outside these criteria, including asymptomatic hyperuricemia, gout not meeting the ACR initiation criteria, and CKD without gout, were classified as documented non-guideline-concordant prescribing. Prescriptions for which no clinical indication was recorded in the electronic medical record were classified as undocumented. This framework intentionally distinguished a documented prescribing decision that did not align with current evidence from the absence of a recorded clinical rationale.

### 2.6. Statistical Analysis

Descriptive statistics were used to summarize patient characteristics, prescribing indications, guideline concordance, and specialty distributions. Age and sex were compared across groups using ANOVA and chi-square tests, respectively. Inferential analyses focused on the two conceptually distinct prescribing-quality outcomes identified a priori: documented non-guideline-concordant prescribing and absence of indication documentation. Specialty-level inferential analyses were restricted to clinical specialties with at least 20 prescriptions to avoid unstable specialty-specific estimates from very small strata. The six eligible specialties were Primary Care, Hematology/Oncology, Cardiology, Nephrology, Rheumatology, and Urology. Lower-volume specialties and prescriptions with no documented prescribing specialty were retained in the descriptive analyses but excluded from specialty-level inferential analyses. The first analysis examined guideline concordance among prescriptions with a documented indication. Univariable binary logistic regression was used to compare documented non-guideline-concordant prescriptions with guideline-concordant prescriptions, with prescribing specialty as the explanatory variable and Rheumatology as the reference category. Cardiology could not be included in the regression model because no Cardiology prescription with a documented indication was guideline-concordant, resulting in complete separation; Cardiology was therefore compared with Rheumatology using Fisher’s exact test. The second analysis examined indication documentation, defined as an undocumented versus documented indication. Because Rheumatology and Urology had no undocumented prescriptions, logistic regression was not appropriate, and pairwise Fisher’s exact tests were used to compare each specialty with Rheumatology. To account for differences in patient characteristics, Firth penalized-likelihood logistic regression models adjusted for age and sex were additionally fitted for both outcomes; Firth regression provides finite estimates in the presence of complete or quasi-complete separation and therefore allowed all six specialties to be retained. Two post hoc sensitivity analyses assessed the effect of undocumented flare history: (1) patients with physician-documented gout but no documented flare history were reclassified as guideline-concordant; and (2) all prescriptions for physician-documented gout were classified as guideline-concordant, irrespective of ACR initiation criteria. Odds ratios (ORs) with 95% confidence intervals (CIs) are reported where estimable. A two-sided *p*-value ≤ 0.05 was considered statistically significant. Analyses were performed using SPSS version 29 (IBM Corp., Armonk, NY, USA).

## 3. Results

### 3.1. Cohort Characteristics and Indication Distribution

Of 1831 patients who received allopurinol from the institutional pharmacy between 1 January 2022 and 30 November 2024, 707 met the eligibility criteria and were included (Figure 1). The mean age was 51.4 years (SD 15.5), and 386 patients (54.6%) were male. Patients with guideline-concordant prescriptions were younger (mean age 47.1 years) and more often male (68.6%) than those with documented non-guideline-concordant (51.0 years; 51.1%) or undocumented prescriptions (55.3 years; 55.3%) (*p* < 0.001 for age; *p* = 0.005 for sex). The most common prescribing context was asymptomatic hyperuricemia in 332 prescriptions (47.0%), followed by no documented indication in 150 (21.2%) and gout not meeting the 2020 ACR ULT initiation criteria in 77 (10.9%). CKD without gout accounted for 43 prescriptions (6.1%), gout meeting the ACR 2020 criteria for 42 (5.9%), TLS management for 40 (5.7%), and recurrent nephrolithiasis for 23 (3.3%). Primary Care was the dominant prescribing specialty (346, 48.9%), followed by Hematology/Oncology (54, 7.6%), Cardiology (48, 6.8%), Nephrology (43, 6.1%), and Rheumatology (31, 4.4%); prescribing specialty was not recorded for 126 prescriptions (17.8%). Complete indication and specialty distributions are shown in Table 1.

### 3.2. Guideline Concordance

Applying the prespecified framework, 105 prescriptions (14.9%) were guideline-concordant, 452 (63.9%) had a documented non-guideline-concordant indication, and 150 (21.2%) had no documented indication. The 105 guideline-concordant prescriptions comprised gout meeting the ACR 2020 criteria (*n* = 42), TLS management (*n* = 40), and recurrent uric acid or calcium oxalate nephrolithiasis meeting guideline-based treatment criteria (*n* = 23). Among the 452 documented non-guideline-concordant prescriptions, asymptomatic hyperuricemia accounted for 332 (73.5%), predominantly in Primary Care (235) and Cardiology (29); gout not meeting the ACR 2020 criteria for 77 (17.0%), predominantly in Primary Care (58); and CKD without gout for 43 (9.5%), predominantly in Nephrology (32) (Table S2). Among the 126 prescriptions without a documented prescribing specialty, 4 (3.2%) were guideline-concordant, 55 (43.7%) were documented non-guideline-concordant, and 67 (53.2%) had no documented indication (Table 2).

**Figure 2:**
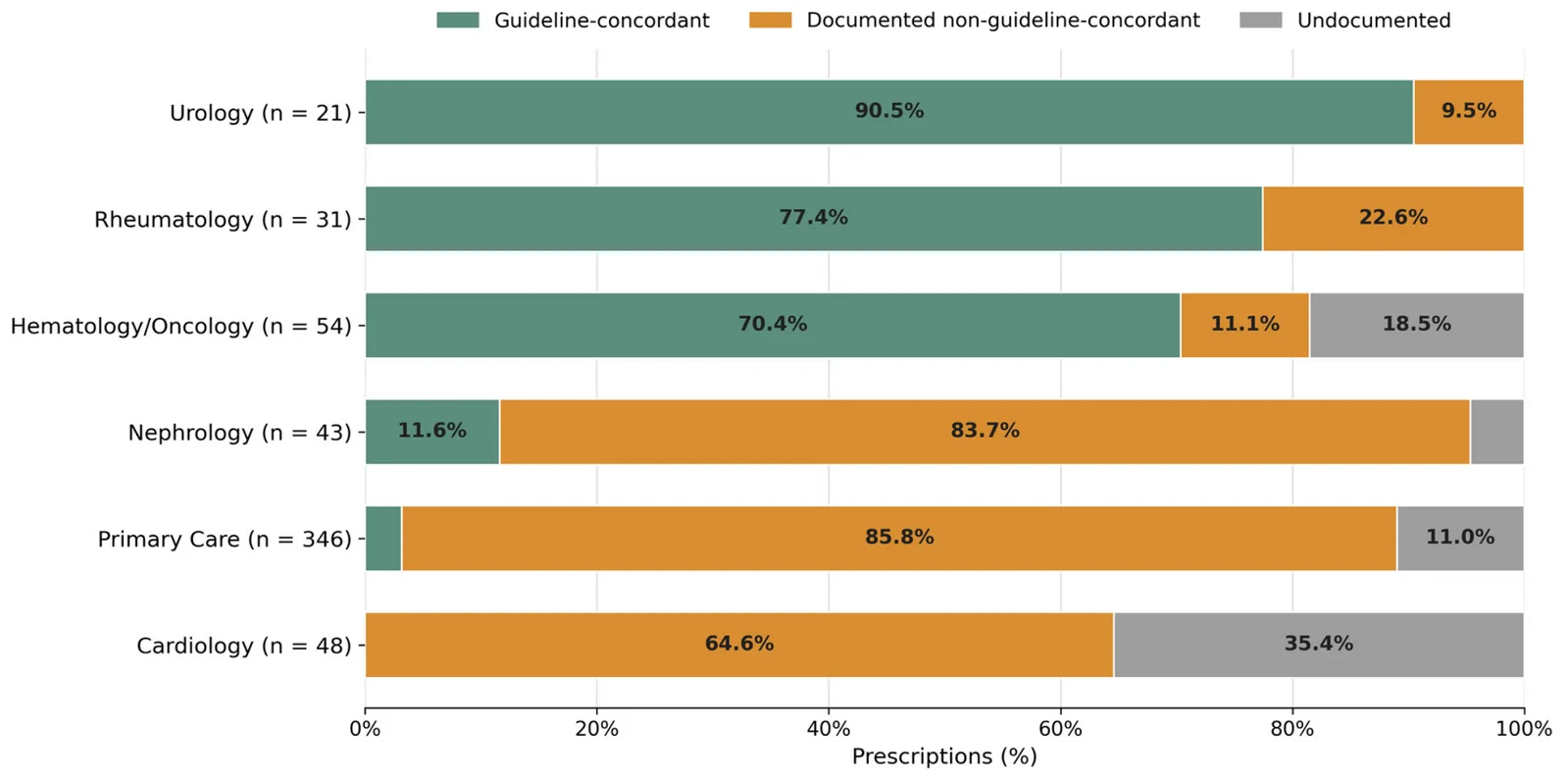
Distribution of guideline concordance and indication documentation across prescribing specialties. Bars show the proportion of allopurinol prescriptions classified as guideline-concordant, documented non-guideline-concordant, or undocumented within each prescribing specialty.

Guideline-concordant prescribing varied substantially across specialties. Urology, Rheumatology, and Hematology/Oncology had the highest concordant prescribing rates (90.5%, 77.4%, and 70.4%, respectively), whereas the corresponding rates were 11.6% in Nephrology, 3.2% in Primary Care, and 0% in Cardiology. The full specialty distribution is presented in Table 2 and illustrated in Figure 2.

### 3.3. Specialty Variation in Guideline Concordance

Among prescriptions with a documented indication, univariable specialty-level analyses demonstrated marked differences in guideline concordance. In univariable logistic regression analysis, Primary Care had substantially higher odds of documented non-guideline-concordant prescribing relative to Rheumatology (OR 92.57, 95% CI 32.89–260.57, *p* < 0.001), as did Nephrology (OR 24.69, 95% CI 7.01–86.90, *p* < 0.001). Hematology/Oncology (OR 0.54, *p* = 0.318) and Urology (OR 0.36, *p* = 0.235) did not differ significantly from Rheumatology.

Cardiology was excluded from the regression model because of complete separation. Among the 31 Cardiology prescriptions with a documented indication, none were guideline-concordant; an additional 17 Cardiology prescriptions had no documented indication. The proportion of documented non-guideline-concordant prescribing in Cardiology was significantly higher than in Rheumatology by Fisher’s exact test (*p* < 0.001). Specialty-level regression results are presented in Table 3. In age- and sex-adjusted models that included Cardiology, Primary Care (adjusted OR 71.37, 95% CI 26.99–211.21), Nephrology (20.04, 6.35–74.36), and Cardiology (144.70, 16.23–19,266.89) remained associated with documented non-guideline-concordant prescribing relative to Rheumatology; age and sex were not independently associated with the outcome (Table 3). In sensitivity analyses, reclassifying patients with gout and undocumented flare history as guideline-concordant increased overall concordance to 22.3% (158/707); Primary Care (OR 49.33, 95% CI 14.43–168.64), Nephrology (OR 54.44, 12.49–237.34), and Cardiology (*p* < 0.001) remained associated with documented non-guideline-concordant prescribing. Classifying all physician-documented gout as guideline-concordant increased concordance to 25.7% (182/707), with similar specialty associations (Table S3).

### 3.4. Specialty Variation in Indication Documentation

Among the six specialties included in the specialty-level documentation analysis (*n* = 543), Rheumatology and Urology had no prescriptions with an undocumented indication. Cardiology had the highest undocumented-indication rate (17/48, 35.4%; *p* < 0.001 versus Rheumatology), followed by Hematology/Oncology (10/54, 18.5%; *p* = 0.012). Undocumented indications occurred in 38 of 346 Primary Care prescriptions (11.0%; *p* = 0.058) and 2 of 43 Nephrology prescriptions (4.7%; *p* = 0.506). In the adjusted model, Cardiology (adjusted OR 31.87, 95% CI 3.91–4147.61), Hematology/Oncology (15.37, 1.84–2005.67), and Primary Care (8.27, 1.11–1056.60) were associated with undocumented indications relative to Rheumatology; the wide confidence intervals reflect the absence of undocumented prescriptions in the reference group. These results are presented in Table 4.

## 4. Discussion

The principal finding of this study is not simply that non-guideline-concordant allopurinol prescribing was common, a pattern already demonstrated in previous Saudi and international studies. Rather, the type of prescribing-quality failure differed markedly across clinical specialties. Primary Care and Nephrology predominantly demonstrated documented prescribing decisions that were inconsistent with contemporary evidence, whereas Cardiology had a substantially greater problem with absent indication documentation. Rheumatology and Urology, in contrast, demonstrated high rates of guideline-concordant prescribing. These findings suggest that allopurinol prescribing-quality gaps within a single health system are heterogeneous and may therefore require different improvement strategies across specialties.

This study adds to the existing literature in three ways. First, it evaluates new rather than prevalent allopurinol use, so the findings reflect current initiation decisions rather than inherited prescriptions. Second, it applies contemporary criteria, including the ACR 2020 guideline and post-2020 trial evidence in CKD, rather than earlier criteria under which marked asymptomatic hyperuricemia could be considered a valid indication [18,19]. Third, and most importantly, it separates two conceptually distinct failures—a documented decision that conflicts with evidence and a prescription with no recorded rationale—and shows that they cluster in different specialties. This distinction is practically useful because the two failures require different interventions, and the framework can be applied to other medicines for which indication-based stewardship is relevant.

Asymptomatic hyperuricemia accounted for 47.0% of all new allopurinol prescriptions and was the dominant source of documented non-guideline-concordant prescribing. This finding is consistent with, but should be distinguished from, previous Saudi reports. Dwid et al. evaluated 1978 allopurinol-treated patients and found that 38.8% were prescribed allopurinol for insignificant hyperuricemia and 39.0% had no documented indication [18]. A subsequent analysis of 594 patients with CKD at the same center found that 76.4% of prescriptions lacked a valid indication; one-third had no documented indication, and when these were grouped by serum urate level, hyperuricemia accounted for 64.2% of prescriptions [19]. Treatment of asymptomatic hyperuricemia is not limited to Saudi Arabia: in a Japanese health insurance claims study, ULT was prescribed to 72.4% of patients identified with asymptomatic hyperuricemia [27], illustrating how local practice norms shape urate-lowering prescribing. The present study therefore does not seek to establish overprescribing as a new phenomenon. Instead, it extends the prior literature by focusing on new prescriptions within the health system, applying contemporary ACR initiation criteria and post-2020 trial evidence, and separating documented guideline discordance from absent indication documentation at the specialty level. Differences in study populations and classification frameworks preclude direct comparison of absolute rates across studies.

The specialty distribution provides additional insight into where prescribing improvement may be needed. Rheumatology and Urology exhibited the highest guideline-concordant prescribing rates, consistent with the close alignment of their clinical scope with established indications for ULT. Primary Care accounted for almost half of all prescriptions (346, 48.9%) but had a guideline-concordant prescribing rate of only 3.2%. Among prescriptions with documented indications, Primary Care was strongly associated with non-guideline-concordant prescribing relative to Rheumatology in univariable analysis (OR 92.57, 95% CI 32.89–260.57). This was not primarily a documentation problem: 85.8% of Primary Care prescriptions carried a recorded indication outside the prespecified criteria, most commonly serum urate elevation documented in the clinical record as the reason for treatment. This pattern is consistent with previously reported knowledge–practice gaps among primary care physicians in Saudi Arabia, including uncertainty regarding ULT initiation thresholds and the distinction between hyperuricemia and clinical gout [20,21,22]. In a recent survey of 744 physicians in Saudi Arabia, fewer than half knew the serum urate threshold that defines asymptomatic hyperuricemia, and general practitioners had the lowest knowledge scores [23]. Our findings extend those survey-based observations by demonstrating a corresponding pattern in real-world prescribing records.

Nephrology accounted for most CKD-without-gout prescriptions (32/43, 74.4%) and was strongly associated with documented non-guideline-concordant prescribing relative to Rheumatology in univariable analysis (OR 24.69, 95% CI 7.01–86.90). This pattern may reflect the historically prevalent hypothesis that lowering serum urate could slow CKD progression. However, the CKD-FIX and PERL trials found no significant renal benefit from allopurinol in patients with CKD or diabetic kidney disease without gout [10,11], and the 2024 KDIGO guideline suggests against urate-lowering therapy to delay CKD progression in asymptomatic hyperuricemia [12]. Continued prescribing for CKD without gout therefore suggests an evidence–practice gap and identifies an opportunity for specialty-focused decision support and education.

None of the 48 Cardiology prescriptions was classified as guideline-concordant: 31 had a documented indication outside the prespecified evidence-based criteria, while 17 (35.4%) had no documented indication. The latter finding is particularly important because the prescribing rationale cannot be reconstructed reliably from the clinical record. One possible explanation is an assumed cardiovascular benefit of urate lowering based on observational associations between hyperuricemia and cardiovascular disease; however, this cannot be established from the present data and remains a hypothesis. Contemporary randomized evidence has not demonstrated cardiovascular benefit from allopurinol in patients without an established urate-lowering indication [28].

The Cardiology pattern also illustrates why undocumented prescribing should not be combined analytically with documented non-guideline-concordant prescribing. Primary Care and Nephrology generally documented the reason for allopurinol use, allowing the evidence–practice gap to be assessed directly. In Cardiology, the higher prevalence of absent indication documentation limits assessment of the underlying clinical reasoning. The first pattern is potentially amenable to evidence-based education and decision support, whereas the second requires more reliable capture of prescribing indications.

These two prescribing-quality patterns suggest that improvement strategies should be matched to the observed problem, rather than applied uniformly across the institution, and support four specific actions. First, for documented non-guideline-concordant prescribing, particularly in Primary Care and Nephrology, focused education should address the distinction between asymptomatic hyperuricemia and clinical indications for ULT, together with dissemination of randomized-trial evidence regarding ULT in CKD without gout. Second, EMR-integrated clinical decision support could prompt prescribers when allopurinol is ordered without a guideline-supported indication [24]; structured referral criteria and interdisciplinary care pathways, as developed for other chronic rheumatic diseases, may also support management of diagnostically uncertain or complex cases [29,30]. Third, for prescriptions without a documented indication, particularly in Cardiology, a structural intervention such as requiring indication capture at the time of prescribing could improve auditability and support subsequent feedback; systematic indication capture in electronic prescribing has been shown to be feasible for pharmacosurveillance [25]. Fourth, periodic audit and feedback using the framework applied here would allow each specialty to monitor progress [31]. These interventions were not tested in the present study and should therefore be regarded as targets for future quality-improvement work.

## 5. Strengths and Limitations

This study has several strengths. Pharmacy dispensing records enabled systematic identification of new allopurinol use within the institutional system over a period of nearly three years, with a look-back of at least five years to exclude prevalent users. A prespecified, guideline-based classification framework distinguished documented non-guideline-concordant prescribing from absence of indication documentation rather than combining them into a single category. The analysis also identified marked specialty-level variation in these two prescribing-quality dimensions, identifying potential targets for subsequent quality-improvement work, and these associations were robust to adjustment for age and sex and to alternative handling of undocumented flare history.

This study has several limitations, which affect its principal findings to different degrees. First, some relate to how the cohort was assembled. The single-center design limits generalizability, and specialty-specific prescribing patterns may reflect the local organization of care. New prescriptions were identified by the absence of any prior allopurinol dispensation in institutional pharmacy records available from September 2016. Prescriptions documented as continuations of treatment initiated elsewhere were excluded, but prior treatment outside the health system could not be completely ruled out. Requiring at least one subsequent KSUMC encounter excluded 34 otherwise eligible patients (4.1% of those reviewed), which is unlikely to have materially affected the findings. Excluding prescriptions with an undocumented specialty and lower-volume specialties from inferential comparisons may also have introduced some selection bias. Second, indication classification depended on information recorded in the EMR, and clinically relevant factors that were not documented may have influenced prescribing decisions. Undocumented prescribing should therefore be interpreted as a failure of documentation, which may coexist with unrecorded but clinically reasonable decisions, rather than as evidence that every such prescription was unjustified. Similarly, patients with gout and no documented flare history were conservatively classified as not meeting flare-based criteria, which may underestimate concordance in this subgroup. However, sensitivity analyses reclassifying these patients did not change the direction or significance of the specialty associations. Third, the specialty comparisons are subject to residual confounding. Analyses were adjusted for age and sex, but clinical covariates such as renal function and comorbidity burden were not systematically abstracted for the full cohort. The associations may therefore partly reflect differences in case mix and should not be interpreted as causal effects of specialty. Sparse outcomes in several specialties also required exact testing or penalized regression, which yielded wide confidence intervals. Finally, the study evaluated prescribing processes rather than clinical outcomes. Adverse drug reactions, disease progression, adherence, and treatment retention were not systematically ascertained, so the safety consequences of non-guideline-concordant initiation could not be quantified and should be examined in prospective studies. Taken together, these limitations bear least on the finding of documented guideline discordance, which rests on the prescriber-recorded indication and explicit criteria. They bear most on the interpretation of undocumented prescribing and of between-specialty differences.

## 6. Conclusions

Guideline-concordant allopurinol initiation was uncommon in this academic health system, and most new prescriptions were issued for indications that current evidence does not support, chiefly asymptomatic hyperuricemia. The central finding is that prescribing-quality gaps are not uniform: documented guideline discordance predominated in Primary Care and Nephrology, whereas absent indication documentation predominated in Cardiology, while Rheumatology and Urology prescribing largely aligned with guidelines. Because these associations are observational and were adjusted only for age and sex, they may partly reflect differences in the patients each specialty manages and should not be attributed to specialty alone. Nonetheless, they indicate that a single institution-wide intervention is unlikely to be sufficient. We recommend evidence-focused decision support and education targeting treatment of asymptomatic hyperuricemia and CKD without gout, mandatory structured indication capture at the time of prescribing, and periodic audit and feedback to monitor progress. Multicenter studies incorporating clinical outcomes, including adverse drug reactions, are needed to confirm these patterns and to evaluate the effect of such interventions.

## Figures and Tables

**Table 1:** Patient characteristics, prescribing indication, guideline concordance, and specialty distribution (*N* = 707).

Characteristic	Value
**Patient characteristics**	
Age, years, mean (SD)	51.4 (15.5)
Male sex, *n* (%)	386 (54.6)
**Documented indication, *n* (%)**	
Gout meeting ACR 2020 ULT criteria	42 (5.9)
Gout not meeting ACR 2020 ULT criteria	77 (10.9)
Asymptomatic hyperuricemia	332 (47.0)
CKD without gout	43 (6.1)
TLS management	40 (5.7)
Recurrent nephrolithiasis	23 (3.3)
**No documented indication, *n* (%)**	**150 (21.2)**
**Guideline concordance, *n* (%)**	
Guideline-concordant	105 (14.9)
Documented non-guideline-concordant	452 (63.9)
Undocumented	150 (21.2)
**Prescribing specialty, *n* (%)**	
Primary Care	346 (48.9)
Hematology/Oncology	54 (7.6)
Cardiology	48 (6.8)
Nephrology	43 (6.1)
Rheumatology	31 (4.4)
Urology	21 (3.0)
Internal Medicine	10 (1.4)
Other specialties	28 (4.0)
Not documented	126 (17.8)
**Total**	**707 (100.0)**

Data are presented as *n* (%) unless otherwise indicated. Guideline-concordant: gout meeting at least one ACR 2020 ULT initiation criterion, tumor lysis syndrome management, or prevention of recurrent nephrolithiasis. Documented non-guideline-concordant: a recorded indication outside the prespecified guideline-based criteria. Undocumented: no indication recorded in the electronic medical record. Other specialties: Endocrinology (5), Orthopedics (4), Emergency Medicine (3), Gastroenterology (3), Gynecology (3), Neurosurgery (3), and one each of General Surgery, Home Healthcare, Hepatopancreatobiliary Surgery, Pain Management, Physical Medicine and Rehabilitation, Pulmonology, and Thoracic Surgery. ACR = American College of Rheumatology; ULT = urate-lowering therapy; CKD = chronic kidney disease; TLS = tumor lysis syndrome; SD = standard deviation.

**Table 2:** Guideline concordance, indication documentation, and patient characteristics by prescribing specialty (*N* = 707).

Prescribing Specialty	Total, *n* (%)	Guideline-Concordant, *n* (%)	Documented Non-Guideline-Concordant, *n* (%)	Undocumented, *n* (%)	Age, Years, Mean (SD)	Male, *n* (%)
Primary Care	346 (48.9)	11 (3.2)	297 (85.8)	38 (11.0)	50.6 (14.2)	166 (48.0)
Hematology/Oncology	54 (7.6)	38 (70.4)	6 (11.1)	10 (18.5)	50.0 (19.1)	30 (55.6)
Cardiology	48 (6.8)	0 (0.0)	31 (64.6)	17 (35.4)	62.7 (15.0)	32 (66.7)
Nephrology	43 (6.1)	5 (11.6)	36 (83.7)	2 (4.7)	49.8 (14.8)	33 (76.7)
Rheumatology	31 (4.4)	24 (77.4)	7 (22.6)	0 (0.0)	45.1 (14.6)	25 (80.6)
Urology	21 (3.0)	19 (90.5)	2 (9.5)	0 (0.0)	46.7 (14.4)	17 (81.0)
Internal Medicine/other specialties	38 (5.4)	4 (10.5)	18 (47.4)	16 (42.1)	53.3 (15.1)	19 (50.0)
Specialty not documented	126 (17.8)	4 (3.2)	55 (43.7)	67 (53.2)	52.1 (16.5)	64 (50.8)
**Total**	**707 (100.0)**	**105 (14.9)**	**452 (63.9)**	**150 (21.2)**	**51.4 (15.5)**	**386 (54.6)**

Data are presented as *n* (%) unless otherwise indicated. Rows are ordered by prescribing volume. Percentages may not sum to 100 because of rounding. Percentages in the Total column are calculated from the full cohort (*N* = 707); percentages in the remaining columns are calculated within each specialty. The “Internal Medicine/other specialties” row combines Internal Medicine (10) and other lower-volume specialties (28). SD = standard deviation.

**Table 3:** Association between prescribing specialty and documented non-guideline-concordant allopurinol prescribing among prescriptions with a documented indication.

Specialty	Unadjusted OR (95% CI)	*p*-Value	Adjusted OR (95% CI) ^†^	*p*-Value
Rheumatology	Reference		Reference	
Primary Care	92.57 (32.89–260.57)	<0.001	71.37 (26.99–211.21)	<0.001
Nephrology	24.69 (7.01–86.90)	<0.001	20.04 (6.35–74.36)	<0.001
Hematology/Oncology	0.54 (0.16–1.81)	0.318	0.45 (0.13–1.50)	0.188
Urology	0.36 (0.07–1.94)	0.235	0.41 (0.07–1.76)	0.232
Cardiology	N/E	<0.001 *	144.70 (16.23–19,266.89)	<0.001
Age (per 10 years)	—		1.21 (0.95–1.56)	0.120
Male sex	—		0.71 (0.31–1.57)	0.392

Unadjusted estimates are from univariable binary logistic regression (*n* = 445 prescriptions with a documented indication), comparing documented non-guideline-concordant with guideline-concordant prescribing, with Rheumatology as the reference category; Cardiology was excluded because all 31 Cardiology prescriptions with a documented indication were non-guideline-concordant, resulting in complete separation. * Cardiology versus Rheumatology by Fisher’s exact test. ^†^ Firth penalized logistic regression adjusted for age and sex, including Cardiology (*n* = 476); profile penalized-likelihood confidence intervals. OR = odds ratio; CI = confidence interval; N/E = not estimable.

**Table 4:** Specialty-level variation in allopurinol indication documentation across the six included specialties (*n* = 543).

Specialty	Undocumented/Total, *n*	Rate, %	*p*-Value *	Adjusted OR (95% CI) ^†^	*p*-Value
Rheumatology (reference)	0/31	0.0	NA	Reference	
Urology	0/21	0.0	1.000	1.44 (0.01–270.47)	0.833
Nephrology	2/43	4.7	0.506	3.70 (0.29–517.14)	0.336
Primary Care	38/346	11.0	0.058	8.27 (1.11–1056.60)	0.035
Hematology/Oncology	10/54	18.5	0.012	15.37 (1.84–2005.67)	0.006
Cardiology	17/48	35.4	<0.001	31.87 (3.91–4147.61)	<0.001
Age (per 10 years)	—			1.08 (0.90–1.28)	0.402
Male sex	—			1.33 (0.77–2.30)	0.304

* Pairwise Fisher’s exact tests comparing undocumented versus documented indication status, with Rheumatology as the reference category; comparisons were exploratory and were not adjusted for multiple testing. ^†^ Firth penalized logistic regression adjusted for age and sex (*n* = 543); the wide confidence intervals reflect the absence of undocumented prescriptions in the reference group. OR = odds ratio; CI = confidence interval; NA = not applicable.

## Data Availability

The data presented in this study are available on request from the corresponding author. The data are not publicly available due to ethical restrictions.

## Supplementary Materials

The following supporting information can be downloaded at: https://www.mdpi.com/article/10.3390/healthcare14203454/s1, Table S1: Guideline-concordance criteria and source guidelines; Table S2: Types of discrepancy by prescribing specialty among documented non-guideline-concordant prescriptions (*n* = 452); Table S3: Post hoc sensitivity analyses for the handling of undocumented gout flare history.
