# Supplementary material for: Guideline Concordance and Documentation of Allopurinol Initiation Across Clinical Specialties: A Three-Year Retrospective Evaluation
Source: Healthcare (Basel). 2026 Oct 15;14(20):3454. doi: 10.3390/healthcare14203454 (PMC13704152; doi:10.3390/healthcare14203454)
Supplement: Supplementary file 1 [file healthcare-14-03454-s001.zip › healthcare-4565377-supplementary.pdf]

Supplementary Materials

Guideline Concordance and Documentation of Allopurinol Initiation Across Clinical Specialties: A Three-Year Retrospective Evaluation

Table S1. Guideline-concordance criteria and source guidelines.

Indication category	Classification	Criterion applied	Source
Gout meeting ACR 2020 initiation criteria	Guideline-concordant	Physician-documented gout with ≥1 subcutaneous tophus, radiographic damage attributable to gout, ≥2 documented flares, or a first flare with CKD stage ≥3, serum urate >9 mg/dL, or urolithiasis	FitzGerald et al., 2020 (ACR) [3]
Tumor lysis syndrome (TLS) management	Guideline-concordant	Allopurinol initiated for TLS prophylaxis or treatment in patients receiving anticancer therapy (prophylaxis recommended across low-, intermediate-, and high-risk categories; treatment of established laboratory or clinical TLS in adults)	Perissinotti et al., 2023 (US expert consensus) [6]
Recurrent nephrolithiasis	Guideline-concordant	Calcium oxalate stones with hyperuricosuria (>4.0 mmol/day), for which allopurinol is first-line treatment; or uric acid or ammonium urate stones in hyperuricosuric stone formers (>4.0 mmol/day)	Skolarikos et al., 2024 (EAU) [7]
Gout not meeting ACR 2020 initiation criteria	Documented non-guideline-concordant	Physician-documented gout without any initiation criterion; patients without documented flare history were classified conservatively as not meeting flare-based criteria	FitzGerald et al., 2020 (ACR) [3]
Asymptomatic hyperuricemia	Documented non-guideline-concordant	Serum urate >6.8 mg/dL documented as the reason for treatment, without documented gout flares, tophi, or another recognized indication	FitzGerald et al., 2020 (ACR; conditional recommendation against ULT) [3]
CKD without gout	Documented non-guideline-concordant	CKD documented by the prescriber as the reason for allopurinol, without physician-documented gout or another guideline-concordant indication	Badve et al., 2020 [10]; Doria et al., 2020 [11]; KDIGO 2024 [12]
No documented indication	Undocumented	No indication recorded in the electronic medical record	—

No hierarchy was required to determine guideline concordance: a prescription was classified as guideline-concordant if at least one guideline-concordant indication was documented. When CKD was documented as the reason for treatment in a patient with hyperuricemia, the prescription was assigned to the CKD-without-gout category. ACR = American College of Rheumatology; CKD = chronic kidney disease; EAU = European Association of Urology; KDIGO = Kidney Disease: Improving Global Outcomes; ULT = urate-lowering therapy.

Table S2. Types of discrepancy by prescribing specialty among documented non-guideline-concordant prescriptions (n = 452).

Prescribing specialty	Asymptomatic hyperuricemia	Gout not meeting ACR 2020 criteria	CKD without gout	Total
Primary Care	235	58	4	297
Cardiology	29	1	1	31
Nephrology	3	1	32	36
Rheumatology	1	6	0	7
Hematology/Oncology	6	0	0	6
Urology	1	0	1	2
Internal Medicine	3	1	2	6
Other specialties	6	4	2	12
Specialty not documented	48	6	1	55
Total, n (%)	332 (73.5)	77 (17.0)	43 (9.5)	452 (100.0)

Data are presented as n. Percentages in the Total row are of all documented non-guideline-concordant prescriptions. Among the 77 patients with gout not meeting the initiation criteria, flare history was not documented in 53, 15 had no documented flare, and 9 had a single documented flare without a first-flare criterion; none had tophi or radiographic damage.

Table S3. Post hoc sensitivity analyses for the handling of undocumented gout flare history.

Analysis	Guideline-concordant, n (%)	Primary Care OR (95% CI)	Nephrology OR (95% CI)	Cardiology vs. Rheumatology
Primary analysis	105 (14.9)	92.57 (32.89–260.57)	24.69 (7.01–86.90)	$p < 0.001$
S1: gout with undocumented flare history reclassified as concordant	158 (22.3)	49.33 (14.43–168.64)	54.44 (12.49–237.34)	$p < 0.001$
S2: all physician-documented gout classified as concordant	182 (25.7)	103.91 (13.92–775.76)	175.00 (19.93–1536.45)	$p < 0.001$

Odds ratios are from univariable logistic regression comparing documented non-guideline-concordant with guideline-concordant prescribing among prescriptions with a documented indication, with Rheumatology as the reference category. Cardiology was excluded from the regression because of complete separation and was compared with Rheumatology using Fisher's exact test. Hematology/Oncology and Urology did not differ significantly from Rheumatology in any analysis. OR = odds ratio; CI = confidence interval.
